# A comparison of a GPS device and a multi-camera video technology during official soccer matches: Agreement between systems

**DOI:** 10.1371/journal.pone.0220729

**Published:** 2019-08-08

**Authors:** Eduard Pons, Tomás García-Calvo, Ricardo Resta, Hugo Blanco, Roberto López del Campo, Jesús Díaz García, Juan José Pulido

**Affiliations:** 1 Sports Performance Area, FC Barcelona, Barcelona, Spain; 2 Department of Didactics of Musical, Plastic and Body Expression, Faculty of Sport Sciences, University of Extremadura, Caceres, Spain; 3 Sports Research Area of LaLiga, Madrid, Spain; 4 Faculty of Human Kinetics. University of Lisbon, Portugal; Nottingham Trent University, UNITED KINGDOM

## Abstract

The aim of this study was to compare the agreement of the movement demands data during a soccer match (total distance, distance per minute, average speed, maximum speed and distance covered in different speed sectors) between an optical tracking system (Mediacoach System) and a GPS device (Wimu Pro). Participants were twenty-six male professional soccer players (age: 21.65 ± 2.03 years; height: 180.00 ± 7.47 cm; weight: 73.81 ± 5.65 kg) from FC Barcelona B, of whom were recorded a total of 759 measurements during 38 official matches in the Spanish second division. The Mediacoach System and the Wimu Pro were compared using the standardized mean bias, standard error of estimate, intraclass correlation coefficients (ICC), coefficient of variation (%), and the regression equation to estimate data for each variable. In terms of agreement between systems, the magnitude of the ICC was *almost perfect* (> 0.90–1.00) for all variables analyzed. The coefficient of the variations between devices was close to zero (< 5%) for total distance, distance per minute, average speed, maximum speed, and walking and jogging, and between 9% and 15% for running, intense running, and sprinting at low and at high intensities. It can be observed that, compared to Wimu Pro the Mediacoach System slightly overestimated all the variables analyzed except for average speed, maximum speed, and walking variables. In conclusion, both systems can be used, and the information they provide in the analyzed variables can be interchanged, with the benefits implied for practitioners and researchers.

## Introduction

The quantification of athletes’ external load has two main objectives: to improve performance and reduce a player’s risk of injury [1, 2]. Hence, the use of technology is an important aid for the analysis of load in sports [3]: (i) to better understand practice sessions (evaluation of demands of any training session or match); (ii) to help program the optimal training load; (iii) and to make decisions about individual players’ training programs [4].

There are different options to quantify team-sport athletes’ external load [5]. The recent exponential advancement of match analysis systems such as semi-automatic multiple-camera video technology (VID), radar-based local positioning system (LPS), and the Global Position System (GPS) has enabled the evaluation of players’ external load [6]. In this study, we shall focus on the comparison of the data obtained by two of these innovative technologies (VID and GPS) during official soccer matches.

VID is a methodology for analyzing external load based on multiple high-definition cameras that track players placed around the soccer field. This system produces the trajectories of players around the pitch throughout the game, and allows researchers with access to the trajectory data to study the movements of individual players and teams and the interactions between them [7].

In recent years, numerous works have analyzed the competition load through the VID system [8, 9]. However, the main problem is VID validity, and for this reason, some researchers suggest that it is necessary to carry out validity studies and compare the degree of agreement of the VID with other assessment systems [10, 11]. In this sense, GPS technology has experienced exponential growth in recent years in its use for the quantification and control of external load in soccer training and matches. This is possible because in 2015, FIFA [12] amended its rules to allow the use of electronic performance and tracking systems (EPTS) in competitive matches [13].

Thus, many investigations have analyzed the capacity of these systems to evaluate conditional variables in training sessions to improve performance or avoid injuries [14, 15]. In addition, recent studies are quantifying these variables in match situations [13, 16, 17] but, to date, we have no knowledge of studies that have compared VID and GPS data in official matches.

Despite this, some investigations have compared the two technologies in training situations or in specific tests [11]. Specifically, these studies have shown that multiple camera semiautomatic systems tend to report slightly-to-moderately [18] and moderately-to-largely [16] greater distances covered at medium and high intensity than GPS technology [10], and demonstrated that ProZone (VID) tended to report greater distances at high speed than the GPS systems. However, most studies have been carried out in non-ecological environments by creating circuits that try to simulate real competition conditions [19], and this may be one of the keys to the results found.

As has been observed, VID and GPS are widely used technologies in research, but researchers have reported the lack of works that compare the results obtained by both methods and have stated that is necessary to continue working to reach high levels of agreement in the conditional parameters between the systems [11, 20, 21, 22]. Therefore, studies that prove the interrelation between these variables with a more ecological perspective are necessary.

Thus, the objective of this work will be the comparison of the Mediacoach conditional variables with the same data obtained from a GPS device (i.e., Wimu Pro). This comparison will be focused on physical parameters. Specifically, the degree of agreement between the two systems in distances covered and speeds will be determined. In addition, a second objective of the study will be to create equations that allow exchanging data between the two models [10].

## Materials and methods

### Participants and procedure

Twenty-six male professional soccer players (age: 21.65 ± 2.03 years; height: 180.00 ± 7.47 cm; weight: 73.81 ± 5.65 kg) from the FC Barcelona B team participated in this study. They had no injuries at the time of data collection. The measurements were recorded in twenty-two different stadiums (depending of the location of the match) during official matches (*n* = 42) of the Spanish second division in the 2017/2018 regular season (LaLiga 123).

To allow data comparison from the two systems and avoid valuation errors, samples were registered from each of the two halves of the matches separately. Specifically, the datasets from the GPS were adjusted with respect to the VID system (i.e., adjusting the GPS recordings according to the VID registers, always at the beginning of each half). In addition, data of some matches were eliminated because, when downloading the raw data from the GPS device or the optical tracking camera, an intermittent signal loss was detected. Specifically, the measurements of the matches belonging to the 29^th^ and 39^th^ rounds were eliminated because these measurement errors were found in the GPS devices (i.e., outlier data in some players), and 5^th^ and 10^th^ rounds were eliminated due to visual technical problems detected in the VID.

The following exclusion criteria were also used for the measurements:

- Players who had a disconnection problem in the GPS device during the game were excluded.
- Goalkeepers were also excluded from the study.

A total of 91 measurements were deleted for these reasons, and the average number of soccer players analyzed per match was 8.88 (SD = 2.17). An ANOVA analysis was also conducted to determine whether the differences between the two procedures used were significantly different among the stadiums where the matches were played. No significant differences were found.

Finally, a total of 759 measurements were recorded (GPS and VID), registering 374 measurements in the first half, and 385 in the second half. In addition, with this reconfiguration technique, the validation error is minimized, facilitating the interpretation of the data [23].

The study received ethical approval from the second author´s university; Vice-Rectorate of Research, Transfer and Innovation—Delegation of the Bioethics and Biosafety Commission (Protocol number: 153/2017). Players received verbal and written information regarding the nature of their voluntary participation in the study. In addition, all participants were treated according to the American Psychological Association ethical guidelines regarding consent, confidentiality, and anonymity.

### Instrumentation

The movement demands data were collected using two different systems: Mediacoach and Wimu Pro.

#### Mediacoach system

The Mediacoach System is a series of super 4K-HDR cameras based on a positioning system (Tracab—ChyronHego VID), which records from several angles and analyzes X and Y positions for each player, resulting in three-dimensional tracking in real-time (tracking data was recorded at 25 Hz per second). Mediacoach is also based on data correction of the semi-automatic VID (the manual part of the process). This correction is made by an overlay of the X Coordinate, provided automatically by the system for each player on the real video image of the match. This detects and visually corrects the situations in which the positioning coordinates are erroneous because they move away from the position of the player to whom the data belong.

#### Wimu Pro

The Wimu Pro device (RealtrackSystems, Almería, Spain) previously validated in other studies [24, 25] was also used for data collection. The device integrates different sensors: four 3D accelerometers operating at different scales: ± 16G, ± 16G, ± 32G and ± 400G; three gyroscopes, two at ± 2000°/s at 1000Hz and one at ± 4000°/s at1000Hz; a 3D magnet ± eight Gauss at 160 Hz; and a barometer ± 1200 mbar at 100 Hz. For the registration of the spatial positioning, speed, and acceleration, the device integrates two sensors: Global Navigation Satellite System and Global Position System GNSS/GPS at 10Hz (numbers of satellites = 8.96; SD = 1.56), compatible with Galileo and Ultra Wave Band (UWB) at 18 Hz; the use of the latter requires the installation of an antenna system around the sports hall. The device has four communication interfaces: WIFI 802.11 b/g/n, Wireless BLUETOOTH, Wireless ANT+, and USB 2.0 (High speed). The recorded data are stored in a 16GB internal flash memory. The device has an internal battery with a four-hour duration, it weighs 70g, and its dimensions are 81x45x16 mm. For collecting, processing, and reporting GPS data, Malone et al.’s [26] applications and considerations were considered (i.e., soccer players should wear the devices in appropriate tight-fitting garments in the upper part of the back to hold the device and minimize unwanted movement; they should also ensure that devices have satellite connection before any data collection [known as GPS lock] and the devices were placed in a clear outdoor space to allow sufficient time to achieve GPS lock).

### Measured variables

Data of the following variables related to movements and speeds were collected:

Total Distance: distance covered in meters (m) by a soccer player during a match, regardless of the position occupied on the pitch.

Distance per minute: distance covered per minute (m) during the time a soccer player participates. It is a relative variable indicating load.

Average Speed: average speed (Km/h) at which the soccer player moves during the game time in which he participates in the match.

Maximum Speed: peak speed (Km/h) reached by a soccer player in a match.

In accordance with Carling [24], distance covered at six different intensities were established:

- *Speed 0–6 Km/h*: Walking.
- *Speed 6–12 Km/h*: Jogging.
- *Speed 12–18 Km/h*: Running.
- *Speed 18–21 Km/h*: Intense running.
- *Speed 21–24 Km/h*: Sprinting at low intensity.
- *Speed more than 24 Km/h*: Sprinting at high intensity.

### Statistical analyses

All statistics were analyzed using IBM SPSS Statistics 24.0 version and Microsoft Excel. First, the descriptive statistics were calculated for both instruments. Bland-Altman plot was created to assess the agreement between methods and determine the systematic bias ± random error and limits of agreement for each variable [27, 28], analyzing the means difference found between the two systems and their limits of agreement (M+/- SD*1.96). Linear regression of the average of the two measures with respect to the means difference were calculated to determine the proportional bias in differences between the methods.

However, as Hopkins pointed [29], this method can be sensitive to small errors and to the size of the sample. For this reason, a linear regression in the data obtained with Mediacoach with respect to Wimu Pro was conducted, and standardized mean bias (SMB) and typical error of the estimate (TEE) were calculated. The magnitude of the effects was evaluated according to [29]. The SMB was rated as *trivial* (< 0.19), *small* (0.2 to 0.59), *medium* (0.6 to 1.19, or *large* (1.2 to 1.99), and the TEE was rated as *trivial* (< 0.1), *small* (0.1 to 0.29), *moderate* (0.3 to 0.59), or *large* (> 0.59) [29]. Also, based on 95% confidence limits (95% CL), the agreement between the criterion measures was assessed using an Excel spreadsheet to calculate the mean bias [29].

Next, the degree of agreement between Wimu Pro and Mediacoach was analyzed using the intraclass correlation coefficient (ICC) and the coefficient of variation (%) of each variable. The following criteria were adopted to interpret the magnitude of the ICC: *trivial* (≤ 0.1), *small* (> 0.10 to 0.30), *moderate* (> 0.30 to 0.50), *large* (> 0.50 to 0.70), *very large* (> 0.70 to 0.90), and *almost perfect* (> 0.90 to 1.00). Finally, the regression equation to estimate Wimu Pro data from Mediacoach data for each variable was calculated.

## Results

Descriptive statistics of each of the variables analyzed by the two instruments, Mediacoach System and Wimu Pro, are displayed in Table 1. It can be observed that, compared to Wimu Pro, the Mediacoach slightly overestimated average distance, distance per minute, jogging, running, intense running and sprinting at low and at high intensity, whereas it underestimated average speed, maximum speed and walking variables.

**Table 1:** Differences between Mediacoach System and Wimu Pro data using Bland and Altman’s method with 95% confidence limits.

Variables	Wimu	Medicoach	Bias	Estimate (SD*1.96)	Lower CL	Upper CL	*r*	*f*^*2*^
*Total Distance*:	4525.7**±**1309.8	4628.7 **±** 1346.5	103.08	167.43	-64.35	270.51	.43	.22
*Distance/Minute*:	107.8**±**18.1	110.1 **±** 18.5	2.34	3.97	-1.63	6.30	.17	.02
*Speed*:	6.8**±**1.0	6.7 **±** 1.1	-0.15	-0.39	-0.53	0.24	.38	.16
*Maximum Speed*:	29.5**±**3.3	29.2 **±** 2.9	-0.39	2.28	-2.66	1.88	.29*	.09
*Walking*:	1453.5**±**417.2	1404,7 **±** 413.2	-48.75	117.25	-166.01	68.49	.06*	.00
*Jogging*:	1589.7**±**578.3	1593.1 **±** 579.7	3.44	99.07	-95.62	102.50	.03	.00
*Running*:	1038.7**±**437.5	1125.6 **±** 466.1	86.92	123.25	-36.33	210.16	.44	.25
*Intense Running*:	228.8**±**106.9	256.2 **±** 118	27.41	45.85	-18.44	73.26	.48	.30
*Sprinting at LI*:	124.1**±**66.4	143 **±** 74.9	18.90	34.57	-15.66	53.47	.48	.30
*Sprinting at HI*:	98.2**±**64.7	114.5 **±** 73.7	16.34	33.15	-16.83	49.50	.57	.46

Notes. LI = Low Intensity; HI = High Intensity.

* = Negative trend.

In accordance with Bland and Altman’s method [27,28], the plots of the all variables were analyzed and systematic errors were found in some variables. The linear regression calculated presents a proportional systematic bias with a positive trend in all the variables, except for maximum speed and distance covered between 0 and 6 km/h (i.e., walking), showing a negative trend in these two variables. Considering the proposal of Cohen [30], the value of *f*^*2*^ for the size of the effect shows that these differences are small or medium, except for the case of sprinting at high intensity. For this reason, we decided to analyze the concurrent validity between the two measures [29], with the aim of clarifying and giving consistency to the results.

As can be seen in Table 2 all SMBs were rated as *trivial* (< .14), and TEEs were rated as *trivial* (< 0.1) in total distance and jogging, and as *small* (0.1 to 0.29) in distance per minute, average speed, walking, running, intense running, sprinting at low and at high intensities. Finally, TEE was rated as *moderate* (0.3 to 0.59) in the maximum speed variable. The magnitude of the ICC was rated as *almost perfect* (> 0.90 to 1.00) for all variables. The coefficient of the variations between devices were close to zero (< 5%) in total distance, distance per minute, average speed, maximum speed, walking and jogging, and between 9% and 15% for running, intense running, sprinting at low and high intensities.

**Table 2:** Comparison of each Variable analyzed by Mediacoach System and Wimu Pro Data, including Standardized Mean Bias, Typical Error of Estimate (TEE), intraclass correlation coefficient (ICC), and coefficient of variation (CV), all with 95% confidence limits.

	Total Distance	Distance–Minute	Average Speed	Maximum Speed	Walking	Jogging	Running	Intense Running	Sprinting at low intensity	Sprinting at high intensity
Standardized Mean Bias	-0.02	-0.01	0.12	0.14	-0.12	-0.01	0.02	0.00	0.02	0.02
95% CLs	[-0.04 to -0.01]	[-0.05 to 0.03]	[0.09 to 0.16]	[-0.02 to 0.31]	[-0.16 to -0.08]	[-0.03 to 0.01]	[0.00 to 0.05]	[-0.03 to 0.03]	[-0.02 to 0.05]	[0.00 to 0.05]
	*Trivial*	*Trivial*	*Trivial*	*Trivial*	*Trivial*	*Trivial*	*Trivial*	*Trivial*	*Trivial*	*Trivial*
Standardized TEE	0.06	0.11	0.17	0.38	0.14	0.09	0.12	0.18	0.22	0.20
95% CLs	[0.05 to 0.06]	[0.10 to 0.12]	[0.16 to 0.19]	[0.35 to 0.41]	[0.13 to 0.16]	[0.08 to 0.09]	[0.12 to 0.13]	[0.17 to 0.20]	[0.21 to 0.24]	[0.18 to 0.21]
	*Trivial*	*Small*	*Small*	*Moderate*	*Small*	*Trivial*	*Small*	*Small*	*Small*	*Small*
ICC	0.99	0.99	0.98	0.94	0.99	0.99	0.98	0.96	0.94	0.97
95% CL	[0.92 to 1.00]	[0.90 to 1.00]	[0.91 to 0.99]	[0.90 to 0.96]	[0.92 to 0.99]	[0.98 to 1.00]	[0.72 to 0.99]	[0.71 to 0.98]	[0.71 to 0.98]	[0.75 to 0.98]
	*Almost Perfect*	*Almost Perfect*	*Almost Perfect*	*Almost Perfect*	*Almost Perfect*	*Almost Perfect*	*Almost Perfect*	*Almost Perfect*	*Almost Perfect*	*Almost Perfect*
% CV	1.8	1.8	2.7	4.0	4.2	3.8	6.8	10.4	13.5	14.9

Notes. CLs = Confidence Limits. Distances covered in different speed sectors: Walking = 0–6 Km/h; Jogging = 6–12 Km/h; Running = 12–18 Km/h; Intense Running = 18–21 Km/h; Sprinting at low intensity = 21–24 Km/h; Sprinting at high intensity = > 24 Km/h.

On the other hand, regarding the proposal to generate an equation that would allow us to validly exchange the data obtained with the two systems, the following procedure was conducted. First, one half of the sample was selected (N = 380) to analyze a linear regression analysis between the two systems. The regression equation to estimate Mediacoach data from Wimu Pro data (Table 3) for each variable is:

Y(Wimudata)=(slope*X(Mediacoachdata))+intercept(residualerrors).

Where Y is the estimated Wimu Pro datum, and X is the Mediacoach datum for a given variable. The intercept represents residual errors in meters (distance variables) or km/h (speed variables). Next, it was compared with the second half of the sample to examine whether the equation adjusted correctly and to determine whether the application of the values to Mediacoach were valid compared with the values of Wimu Pro.

**Table 3:** The regression equations for each variable in the study.

Variables	Equations	ICC	Bias	SMB	TEE
*Total Distance*	y = 0.971x + 29.653 m	.999	-5.20	0.00	0.06
*Distance/Minute*	y = 0.977x + 0.115 m	.998	-.05	-0.01	0.10
*Speed*	y = 0.914x + 0.770 m/s	.992	.05	0.03	0.17
*Maximum Speed*	y = 1.041x − 0.787 m/s	.966	.05	0.01	.28
*Walking*	y = 1.002x + 35.458 m	.995	-9.92	-0.04	0.14
*Jogging*	y = 0.988x + 14.036m	.998	-.77	0.01	0.09
*Running*	y = 0.927x − 3.756 m	.996	.76	0.01	0.12
*Intense Running*	y = 0.892x + 1.055 m	.991	.71	0.00	0.19
*Sprinting at LI*	y = 0.869x + 0.034 m	.988	.10	0.00	0.22
*Sprinting at HI*	y = 0.858x + 1.059 m	.989	1.12	0.02	0.21

Notes. LI = Low Intensity; HI = High Intensity.

## Discussion

The main aim of this study was to compare two systems designed and developed to measure the movement demands of soccer. Using two different methods [27, 29], and considering the terms of agreement between the systems, similar data were obtained from the Mediacoach System and from the Wimu Pro in the total distance covered, distance per minute traveled, average and maximum speed and covered distance in several speed sectors. These findings are in line with those reported by other authors in different studies focused on analyzing the movement loads during soccer matches [18]. In this sense, very similar results were registered in the total distances covered and the distances traveled in different speed intervals recorded by the two systems compared to other results found in previous research [10].

In according to the results obtained in this study, we highlight that the Mediacoach System systematically overestimated the scores of the distance variables, except for distance covered at 0–6 km/h, compared to the Wimu Pro. Conversely, average speed and maximum speed variables (i.e., variables analyzed in Km/h) reached higher values with the Wimu Pro system than with Mediacoach. Also, after analyzing the values of the regression of the averages with respect to the means differences, a positive systematic error was found in all the variables examined, except for the maximum speed and the distance covered between 0 and 6 km/h (i.e., walking). These results are consistent with the findings of most previous studies [31] concluding that both GPS technology and the computer-based tracking system involve systematic errors, overestimating the distance traveled, although this is the first study focused on evaluating performance indicators with professional soccer players during official matches and in a full season.

We emphasize that these errors are relatively small and predictable, so it is considered that the use of either of these technologies should be promoted in order to monitor players’ movements. Thus, the standardized mean bias was *trivial* for all variables, with a *trivial* and *small* TEE, except for maximum speed, where TEE was *moderate*. The ICCs between the two systems were *almost perfect* in all the analyzed variables.

Other studies have shown that multiple camera semiautomatic systems tend to report slightly-to-moderately [18] and moderately-to-largely [32] greater distances covered at medium and high intensity than GPS technology. In these investigations, results highlighted that all the systems similarly detected the fatigue produced during the match, and there were differences between the instruments in the estimation of the distances traveled in each of the speed categories. Greater total distances were recorded (around 1 km) with VID and 5-Hz GPS systems than with 1 Hz GPS devices, and the video system [10] demonstrated that, whereas there were small between-system differences in total distance, ProZone (VID) tended to report greater distances at high speed than the other three systems.

Linke, Link, and Lames [11] also assessed the measurement accuracy of the most commonly used tracking technologies in professional team sports (i.e., semi-automatic multiple-camera video technology, LPS and GPS), concluding that differences between technologies were not as pronounced in distances and speeds, but all technologies had in common that the magnitude of the error increased as the speed of the tracked object increased. Results revealed technology-dependent accuracy variations in the video tracking system. As the movement direction of the shuttle run was conducted on the vertical (perpendicular) camera axis (Y-axis), video tracking tended to overestimate the peak speed during shuttle runs. In our case, differences between the two systems were smaller than in previous studies, possibly due to the technological advances of the two information collection procedures. In recent years, a huge effort has been made to improve GPS technologies [24, 33] and to increase the number of cameras to have greater coverage of the stadium from more angles and to improve the resolution quality of the cameras to automatically detect and track each player by his identification number [34].

On another hand, most studies have been carried out in non-ecological environments by creating circuits that simulate real competition conditions [19]. However, it is interesting to note that this is the first time that a VID is compared with real tracking data during official matches using a GPS device. Moreover, we highlight that the measurements were taken in very different environmental situations, stadiums in different geographical locations at different times, compared to other studies that have been carried out in a single stadium at a specific time [35].

Another very interesting finding of this research is the development of predictive equations that allow interchanging data from the two different systems. In this line, Buchheit and Simpson [4] consider that the ideal system does not yet exist, and that all systems have advantages and disadvantages. To allow for an adequate evaluation of a player’s overall movement load, and to integrate the data from different systems accordingly, practitioners are recommended to use calibration equations. In our case, we have confirmed the equations that allow us to determine the distances and speeds that players would reach in the Wimu Pro when collected through the Mediacoach System or vice versa. Few researches have been able to generate this equation with a referent sample and from an ecological perspective. This implies added system interchangeability as an important point for practitioners in professional clubs, who often use two different systems over the week [10].

### Limitations and practical applications

Some limitations were detected during the development of this study. Despite comparing some movement demands in the two systems to understand players’ workload during the match, it would also have been interesting to analyze accelerations and decelerations variables [4]. Finally, we point out that the systems used are not totally accurate, so it would be interesting to compare them with the VICOM system to determine the degree of agreement [11].

The development of this kind of study can also generate several interesting benefits and practical applications for professionals and researchers. Firstly, the use of these advanced approaches furthers our understanding of specific position work-rate profiles of soccer players and their fitness requirements, the intensities of discrete activities during the match, and the occurrence of a reduced work-rate among players [36]. In addition, knowledge of these variables related to players’ physical load volume and intensity during the match can be provided from a scientific perspective in order to better understand game situations, improve task training from these situations, and design models that allow us to improve performance or prevent injuries, and optimize the control and quantification of loads for the individualization of training [4].

On another hand, as we have established the equations that allow interchanging data between the two systems analyzed, many clubs could benefit from this. The multi-camera video Mediacoach System is a non-intrusive method, avoiding various problems that may arise in the players. Also, this study can resolve the problems that the GPS appears to have in certain stadiums or climatic conditions that block the signal, and data from competitions can be obtained that can be used later by coaches and researchers. However, and taking into account all the above, we are aware that we must be cautious about the interchangeability of the data and continue to improve the accuracy of GPS. In the future, this could be applied to other systems of analysis.

In this line, the Mediacoach System also allows analyzing technical-tactical behaviors (e.g., the specific position of the ball) that occur during the game, or players’ specific technical actions, a matter that has been considered essential in the latest contributions of performance analysis [3, 37]. Many authors have proposed the need to integrate all these variables to gain complex knowledge of individual and collective behavior during matches, which can be used to make objective decisions to structure the training elements and for subsequent match preparation [22, 23, 38].

Finally, as the Mediacoach System registered the last 10 seasons in the LaLiga competitions (First and Second Division), studies that allow us to longitudinally compare the evolution of the game demands can be performed. This can help coaches and researchers to improve their knowledge of the game of soccer and enable closer monitoring of aspects such as the evolution of the physical demands over time [8]. In addition, this device will enable us to analyze specific performance demands related to other variables such as high speed running distance, high metabolic load distance, acceleration and deceleration… even considering different match situations.

To sum up, we highlight the advantages and disadvantages of using the two systems to determine in greater depth the characteristics and demands of this sport in real situations. GPS contributes precision, immediacy and informational richness about external and internal load [22], but it also presents some problems in the estimate measurements [11], and cannot be used with court-based sports held indoors, due to the lack of satellite reception [39]. On another hand, VID is a technology which does not require players to have any equipment attached to them and allows researchers with access to the trajectory data to study movements of individual players and teams and the interactions between them [8]. However, VID does not allow access to internal variable information. Due to all these issues, we consider that these technologies are not opposed and that they offer many possibilities of collaboration.

## Conclusion

This study reveals a good agreement in the comparison of two systems designed to analyze each player’s movement demands in professional soccer performance. Specifically, the Mediacoach System slightly overestimates the variables analyzed in meters such as total distance covered, distance per minute, and distance covered in several speed sections (except for *walking*), whereas the Wimu Pro overestimates variables measured in Km/h such as average speed and maximum speed. Therefore, these data provide high ecological relevance to analyze real match situations related to physical demands. In addition, the bases have been established to generate predictive equations that allow exchanging data with other analytical devices, with the benefits that this can imply for practitioners and researchers.

### Permissions statements

The authors declare that all appropriate permissions have been obtained from Mediacoach System and Wimu Pro for the use of these systems in this research and in subsequent publications.

## Supporting information

S1 File Data Mediacoach and Wimu.xls. (XLS)
